# Cheetah-MS: a web server to model protein complexes using tandem cross-linking mass spectrometry data

**DOI:** 10.1093/bioinformatics/btab449

**Published:** 2021-06-15

**Authors:** Hamed Khakzad, Lotta Happonen, Johan Malmström, Lars Malmström

**Affiliations:** Equipe Signalisation Calcique et Infections Microbiennes, École Normale Supérieure Paris-Saclay, Gif-sur-Yvette 91190, France; Institut National de la Santé et de la Recherche Médicale U1282, Gif-sur-Yvette 91190, France; Division of Infection Medicine, Department of Clinical Sciences, Lund University, Lund SE-22184, Sweden; Division of Infection Medicine, Department of Clinical Sciences, Lund University, Lund SE-22184, Sweden; Division of Infection Medicine, Department of Clinical Sciences, Lund University, Lund SE-22184, Sweden

## Abstract

**Summary:**

Protein–protein interactions (PPIs) are central in many biological processes but difficult to characterize, especially in complex, unfractionated samples. Chemical cross-linking combined with mass spectrometry (MS) and computational modeling is gaining recognition as a viable tool in protein interaction studies. Here, we introduce Cheetah-MS, a web server for predicting the PPIs in a complex mixture of samples. It combines the capability and sensitivity of MS to analyze complex samples with the power and resolution of protein–protein docking. It produces the quaternary structure of the PPI of interest by analyzing tandem MS/MS data (also called MS2). Combining MS analysis and modeling increases the sensitivity and, importantly, facilitates the interpretation of the results.

**Availability and implementation:**

Cheetah-MS is freely available as a web server at https://www.txms.org.

## 1 Introduction

Cross-linking mass spectrometry (XL-MS) is a powerful technique to measure protein–protein interactions (PPIs) directly in complex samples (O’Reilly *et al.*, 2018). Bi-functional reagents are used to covalently link two specific residues when the proteins are in their native states. The proteins then undergo enzymatic digestions resulting in many peptides linked by the reagents. The length of the crosslinker arm reveals the maximum distance between the two cross-linked amino acids, and this information is then used to identify and characterize the PPI. Using macromolecular modeling tools such as Rosetta (Koehler *et al.*, 2020), a structural model can be created if enough cross-linked peptides are identified. Here, we propose Cheetah-MS, a web server based on our previously published method, targeted chemical cross-linking MS (TX-MS), a deep integration of protein structure modeling, and chemical XL-MS (Hauri *et al.*, 2019). The power of Cheetah-MS relies on its fast convergence to the solution due to iterative sampling and filtering by XL peptides, where we reduced the number of decoy sampling by order of magnitude. Cheetah-MS supports tandem MS/MS acquisition data type based on non-cleavable reagents (DSS/BS3, DSG and EGS) and can detect up to 12 post-translational modifications (PTMs). 

## 2 Implementation

**Fig. 1.**
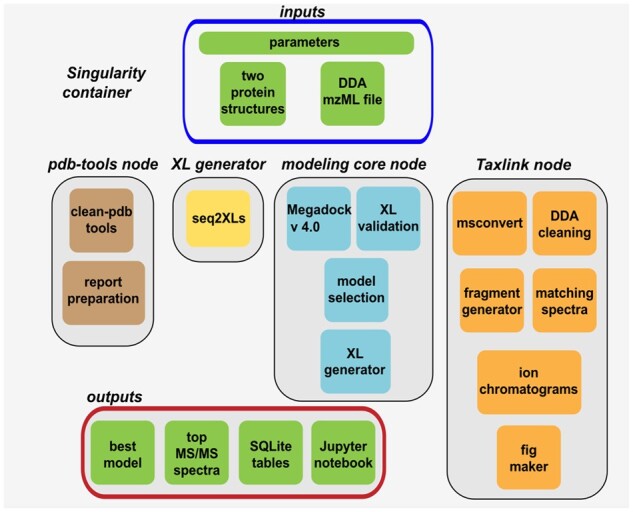
The computational workflow of Cheetah-MS. A singularity container is responsible for managing the workflow and the report system

Cheetah-MS is implemented using applicake (a python package), making the whole workflow easy to connect and flexible for further development. It is composed of four main applicake nodes, including PDB-tools, XL-generator, modeling-core and Taxlink (Fig. 1). The first node uses PDB-tools (Rodrigues *et al.*, 2018) to clean up the input PDBs, recognize the chains, retrieve the sequences and combine the two PDBs into a starting conformational model. XL-generator provides a complete list of all theoretical XLs without considering distance cutoff. Next, this list is passed to Taxlink for MS/MS data analysis. In case the input file is not already in Mascot Generic Format, msconvert from ProteoWizard (Kessner *et al.*, 2008) converts the input mzML file to MGF file format. This file goes then for a filtering/cleaning process according to the XLs provided by the previous step where only spectra containing the monoisotopic mass/charge of interest are passed to the filtered version of the file. Here, for each XL, a set of ion fragments are produced, and their pattern is investigated through the filtered MGF file to find the match. In the modeling-core, selected XLs from the Taxlink node are used to score a set of docking models (2000 models for all runs), provided by Megadock v4.0 (Ohue *et al.*, 2014), and the top scored models are selected. Finally, the best model that supports the largest number of XLs is chosen to be visualized in the output.

To run Cheetah-MS, users need to provide two PDB files and one MS/MS mzML (or converted MGF file) containing the XL-MS data. The advanced options to set include the XL agent, the PTM(s) of interest, the number of final models, the cutoff threshold for modeling, the delta-window for precursor and product ion detection, and finally, the intensity value to remove the background noise in MS/MS data analysis.

After submitting the workflow, the status of the running job is shown, containing the job identifier at the top and the exact processing time of each submodule below. Once the workflow is finished, the best-scoring model is visualized using the NGL viewer (Rose *et al.*, 2018) together with the data analysis report in a Jupyter Notebook. The report was designed to both allow a user to assess the results quickly and to download and extend them to gain deeper insights, often in project-specific ways.

## 3 Results and applicability

Cheetah-MS has been applied to several case studies as the core MS/MS analysis part of the TX-MS approach. Table 1 summarizes the list of published studies where Cheetah-MS was applied for MS/MS data analysis. Also, to test the applicability of the workflow in the webserver context, we reconstructed the *Streptococcus pyogenes* M1 protein interactions with two human plasma proteins (fibrinogen and albumin) based on MS/MS samples obtained from recombinant M1 protein and purified human plasma fibrinogen and albumin. This has resulted in 27 and 10 XLs between M1-fibrinogen and M1-albumin, respectively. Based on the list of detected XLs and produced models, the same binding interface is obtained compared to the initial study (details on the web server manual page).

**Table 1.** The applicability of Cheetah-MS as the core MS/MS analysis of the TX-MS approach in several case studies

Study	Partner proteins	# XLs
GAS M1 protein’s interactome (Hauri *et al.*, 2019)	M1, fibrinogen, albumin, haptoglobin, SerpinA1, coagulation factor XIII A, C4BPa and IgG1	204
Membrane attack complex (Khakzad *et al.*, 2020)	Complement proteins: C5b, C6, C7, C8 and C9	126
GAS M1 interaction with human IgGs (Khakzad *et al.*, 2021)	M1, IgG1, IgG2, IgG3 and IgG4	21
Structure determination of Dermatan sulfate epimerase 1 (Hasan *et al.*, 2021)	DS-epi1	24
GAS M28 interaction with human IgAs (Chowdhury *et al.*, 2021)	M28, IgA1, IgA2 and C4BP	14

## Funding

This work was supported by the Foundation of Knut and Alice Wallenberg [2016.0023 and 2019.0353 to J.M. and L.M.] as well as Vetenskapsrådet 2020-02419 to L.M., and by the Swiss National Science Foundation [P2ZHP3_191289 to H.K.].

*Conflict of Interest*: none declared.
